# Interventions following a high violence risk assessment score: a naturalistic study on a Finnish psychiatric admission ward

**DOI:** 10.1186/s12913-016-1942-0

**Published:** 2017-01-11

**Authors:** Jenni Kaunomäki, Markus Jokela, Raija Kontio, Tero Laiho, Eila Sailas, Nina Lindberg

**Affiliations:** 1 Institute of Behavioural Sciences, University of Helsinki, Siltavuorenpenger 1A, P.O. Box 9, 00014 Helsinki, Finland; 2 Helsinki University and Helsinki University Hospital, Psychiatry, Välskärinkatu 12 A, 00029 HUS Helsinki, Finland; 3 Kellokoski Hospital, 04500 Kellokoski, Finland; 4 Helsinki University and Helsinki University Hospital, Forensic Psychiatry, Välskärinkatu 12 A, 00029 HUS Helsinki, Finland

**Keywords:** Aggression, The dynamic appraisal of situational aggression, Intervention, Violence risk assessment

## Abstract

**Background:**

Patient aggression and violence against staff members and other patients are common concerns in psychiatric units. Many structured clinical risk assessment tools have recently been developed. Despite their superiority to unaided clinical judgments, staff has shown ambivalent views towards them. A constant worry of staff is that the results of risk assessments would not be used. The aims of the present study were to investigate what were the interventions applied by the staff of a psychiatric admission ward after a high risk patient had been identified, how frequently these interventions were used and how effective they were.

**Methods:**

The data were collected in a naturalistic setting during a 6-month period in a Finnish psychiatric admission ward with a total of 331 patients with a mean age of 42.9 years (SD 17.39) suffering mostly from mood, schizophrenia-related and substance use disorders. The total number of treatment days was 2399. The staff assessed the patients daily with the Dynamic Appraisal of Situational Aggression (DASA), which is a structured violence risk assessment considering the upcoming 24 h. The interventions in order to reduce the risk of violence following a high DASA total score (≥4) were collected from the patients’ medical files. Inductive content analysis was used.

**Results:**

There were a total of 64 patients with 217 observations of high DASA total score. In 91.2% of cases, at least one intervention aiming to reduce the violence risk was used. Pro re nata (PRN)-medication, seclusion and focused discussions with a nurse were the most frequently used interventions. Non-coercive and non-pharmacological interventions like daily activities associated significantly with the decrease of perceived risk of violence.

**Conclusion:**

In most cases, a high score in violence risk assessment led to interventions aiming to reduce the risk. Unfortunately, the most frequently used methods were psychopharmacological or coercive. It is hoped that the findings will encourage the staff to use their imagination when choosing violence risk reducing intervention techniques.

## Background

Patient aggression and violence against staff members and other patients are common concerns in psychiatric units [1, 2]. In a Finnish nationwide interview study, as many as 8% of psychiatric staff reported experiencing violence from patients at least once a week and 16% one to three times per month [2]. In a study by Ross et al. [3], 21% of patients had experienced an aggressive incident with another patient during their first 2 weeks of hospitalization. Patient aggression affects the physical and psychological health of personnel [4, 5], and the fear generated from working in a climate of potential danger can undermine patient care [6]. The patients who have acted aggressively are often subject to restrictive interventions, including restraint and seclusion [7]. However, using these strategies may increase even more stress and tension on the ward [8]. Against this background, efforts have been stepped up to increase the active use of de-escalation interventions and to reduce coercive measures in managing of patients’ aggression and violence [7, 9, 10].

Violence management is a key component when working with potentially violent patients. By using a list of empirically supported risk factors, the staff may identify patients’ behavior which could possibly trigger upcoming aggressive events and most importantly, prevent incidents of aggression [11–14]. Identifying empirically supported risk factors also provides the staff more time to prepare themselves for the forthcoming event, or to prevent these events by means of specific interventions [11]. Traditionally, clinicians have used unstructured clinical risk assessment methods (e.g., the hospitals’ own checklists) – with varying degrees of effectiveness [15]. Recently, a number of structured clinical risk assessment tools have been developed and they have proved to be substantially more accurate than unaided clinical judgments [15–17].

Despite the predictive accuracy of structured violence risk assessments, personnel have shown mixed and ambivalent views towards them [18–20]. Insufficient training, a lack of organizational support, costs as well as a lack of multidisciplinary collaboration have been mentioned as hindrances to their implementation [20–22]. Further, research about the utility of structured violence risk assessments at the grassroots is still limited, awakening an ongoing debate around the usefulness and purposefulness of these tools in everyday nursing [20]. One of the problems in the implementation of violence risk assessments in psychiatric wards has been the nursing staff’s concern that the results of the assessments would not be used [20]. This is understandable, since the whole risk assessment procedure is obviously worthless if it does not lead to interventions aiming to reduce the high risk observed.

The aims of the present study was to investigate what were the risk-decreasing interventions applied by the staff on a psychiatric ward after a high risk patient was identified, how frequently these interventions were used and how effective they were.

## Methods

### The psychiatric admission ward

The data were collected in a naturalistic setting between 1.5.-31.10.2013 in an acute psychiatric admission ward in Southern Finland. This ward for adults (≥18 years) with 18 beds serves a catchment area of 185, 000. The ward staff includes two psychiatrists, nursing staff (20.5 positions) and the services of a psychologist, social worker, occupational therapist and ward secretary. During the study period, altogether 331 patients with a mean age of 42.9 (SD 17.39, range 18–90, median 42) were admitted to the ward with a total of 427 treatment periods due to multiple periods by some of the patients. A total of 163 treatment periods (38.2%) started involuntarily. The mean duration of a treatment period was 6 days (SD 6.50, range 1–44, median 4). The total number of hospital days was 2399, and the average occupancy rate of the ward was 74%. According to the Hospital District’s Risk Report System (an electronic management tool used by the staff, which produces statistics and reports about deviations at the hospital’s everyday life), during the study period, altogether 22 incidents of violence occurred on the index ward: eight incidents included physical and 12 psychological violence (verbal assaults and/or threatening situations, which cause fear and insecurity among patients and/or staff) as well as two incidents targeted towards property.

### The Dynamic Appraisal of Situational Aggression (DASA)

The Dynamic Appraisal of Situational Aggression (DASA) is a structured violence risk assessment to be used in a clinical ward setting to identify acute risk of patient aggression within 24 h of the assessment [17]. It consists of seven items (irritability, impulsivity, unwillingness to follow directions, sensitivity to perceived provocation, easily angered when requests are denied, negative attitudes, and verbal threats) of which the occurrence during the previous 12 h is estimated on a two-point scale (0 = absent, 1 = present). The estimations are summed up to give a total score from zero to seven describing the violence risk. The total scores are divided into three categories: 0, 1–3, and 4 or more representing no risk, moderate risk, and high risk, respectively. DASA has proven to be useful in a non-forensic clinical environment [23] with predictive validity ranging from moderate to strong [16, 18, 23]. In the present study, the Finnish version of the instrument was used. It was prepared by a subgroup of authors (TL, ES, NL) using the iterative process of translation and independent back translation, followed by a discussion to resolve minor differences.

### Scoring procedure

A comprehensive coaching session was held for the staff of the ward followed by a 2-week pilot phase in order to practice the use of the violence risk assessment. The inpatients were scored daily. The ratings were completed separately for each patient at 1 pm during the afternoon ward report. Nursing staff on duty filled in the DASA assessments with psychiatrists present when available. So, the assessments represented consensus statements.

### Interventions following a high DASA total score

According to Ogloff and Daffern [17], a DASA score of 0 suggests that the risk of violence is very low, scores of 1–3 suggest that the risk of violence is moderate; and scores of 4 or more indicate that the risk of violence is high. As the goal of using DASA is not just to predict risk for aggression but to help manage the risk, scores of 4 or more should be considered high enough to require immediate intervention aimed to reduce the likelihood of patients engaging in physical aggression [17]. On this basis, the DASA total score with a cut-off point of 4 was used in the present study. To gather up the interventions used after a high score (during the next 24 h after the index score), the hospital files of all patients with DASA total scores ≥4 were reviewed by two independent researchers (a doctor and a nurse) using inductive content analysis, which is a research method for systematic and objective analysis of documents [24]. First, patients’ medical files were read through several times. The unit of analysis was an utterance, which could be a sentence or part of a sentence consisting of thematic content relevant to the research question. Second, reduction of the data was done by picking out and underlining phrases answering the research question. Third, data was coded by labeling reduced phrases with a description according to thematic content that could be seen to characterize the phrases. In the fourth phase, subcategories were formed for these coded phrases by grouping together those with similar content. Any outstanding discrepancies concerning subcategories were resolved. Finally, the main categories were established by grouping together subcategories with similar meaning.

Later, applied interventions were clustered into four groups (interventions regulated by the Finnish Mental Health Act, pro re nata (PRN) - medication, discussion with a nurse, and other interventions (the list of various interventions is presented in Table 2). The DASA total scores between an intervention day and the subsequent day were compared to examine the effectiveness of each intervention group. The frequency and percent distribution of interventions and intervention groups were calculated.

### Statistical analysis

The Chi-square analysis was used to compare distributions of gender and primary diagnoses between the patients with at least one high total score (DASA ≥4) during his/her treatment period and those with only low total scores (DASA <4). Respectively, age was compared between the groups using the Mann-Whitney U-test. The same comparisons were performed between males and females with high DASA total scores. To study the associations between the interventions and the violence risk after the intervention had been applied, we first created a change score indicating the change on DASA scores between the intervention day and the following day (the DASA score on the following day minus the DASA score on the intervention day). We then predicted this change score with five multilevel linear regression models: four models to study the separate associations of the four intervention categories with the DASA change score, and the fifth model to study the associations when all interventions were mutually adjusted for the use of other interventions. The multilevel model took into account the non-independence of the repeated measurements from the same participants over time, providing correct estimates for the standard errors. Baseline DASA total score was adjusted in all the models. Statistical analyses were carried out using the IBM SPSS Statistics version 22.

### Ethics

The study plan was evaluated by the Ethics Committee of the Helsinki and Uusimaa Hospital District. Permission to conduct the study was granted by the administration of the Helsinki and Uusimaa Hospital District. The study was performed in accordance with the Declaration of Helsinki.

## Results

### Characteristics of the sample

Altogether 331 individuals were admitted to the ward during the study period. Most of the patients were hospitalized only once, but some of them had more than one treatment period on the ward during the study period (two treatment periods: *n* = 25; three: *n* = 15; four or more: *n* = 7). Thirty-one (9.4%) patients were discharged from the ward or transferred to another ward before the afternoon DASA scoring. Thus, the DASA assessment was completed at least once with 300 inpatients (males: 49%). The complete description of patients’ principal diagnoses given by the specializing physicians and verified by the deputy chief physician, a specialist in psychiatry, is presented in Table 1. The diagnoses were collected from the patients’ current medical case summaries. According to the International Classification of Diseases (ICD-10) [25], most frequent principal diagnoses of the patients were mood disorders (*n* = 123, 41.0%) and schizophrenia-related disorders (*n* = 106, 35.3%) followed by substance use disorders (*n* = 28, 9.2%). In total, 2193 violence risk assessments were performed. Of 300 patients, 64 (21.3%) (35 men and 29 women) scored ≥4 at least once during their hospital treatment.

**Table 1:** The principal diagnoses according to the ICD-10 in 300 inpatients with the Dynamic Appraisal of Situational Aggression (DASA) scorings

	Number	Percent
F0-09 Organic, including symptomatic, mental disorders	10	3.3
F10-19 Mental and behavioral disorders due to psychoactive substance use	28	9.3
F20-29 Schizophrenia, schizotypal and delusional disorders	106	35.3
F30-39 Mood disorders	123	41
F40-49 Neurotic, stress related and somatoform disorders	14	4.7
F50-59 Behavioral syndromes associated with physiological disturbances and physical factors	2	0.7
F60-69 Disorders of adult personality and behavior	7	2.3
F70-79 Mental retardation	2	0.7
F80-89 Disorders of psychological development	2	0.7
F90-98 Behavioral and emotional disorders	1	0.3
F99 Unspecified mental disorder	5	1.7
	300	100

The gender distribution (X^2^ = 0.993, *p* = NS) and mean age (U = 7500.00, *p* = NS) did not significantly differ between the patients scoring a high DASA total score (≥4) at least once during the treatment period and those exhibiting only low total scores (<4). The patient group with at least one high DASA total score comprised significantly more patients with organic brain disorders (F00-09) (X^2^ = 14.599, *p* < 0.001), and, significantly less patients with mood disorders (F30-39) (X^2^ = 11.749, *p* < 0.001). Focusing on 64 patients with at least one high DASA total score, no significant gender differences were observed in age or primary diagnoses. Among these 64 patients, the total number of hospital days with a high DASA total score ranged from 1 to 18 (median 3). Most of the patients had 1 to 3 hospital days with a high DASA total score, but there were 17 (26.7%) patients who were ranked above the median.

### Interventions following a high DASA total score

Sixty-four patients had altogether 217 observations with a DASA total score ≥4. At least one intervention was followed in 198 of 217 observations (91.2%). In 19 (8.8%) cases, no intervention was followed. A total of 400 interventions were reported. The average amount of interventions followed after a high DASA total score was 1.8 (SD = 1.1, median = 2). The most frequently used interventions were PRN-medication, seclusion and focused discussion with a nurse. The complete list of all reported interventions and the number of individuals targeted with each of these interventions are presented in Table 2. The usage of altogether 106 (26.3%) interventions was legitimized by the Finnish Mental Health Act, which means that these interventions can only be used if the patient is detained in involuntary treatment or under observation.

**Table 2:** Interventions following a high violence risk assessment score (the Dynamic Appraisal of Situational Aggression [DASA] total score ≥4)

Observations with a high violence risk assessment score	*n* = 217		
	n	%	
No following interventions	19	8.8	
One or more interventions	198	91.2	
A total number of observed interventions	*n* = 400		
Distribution of various interventions	n^I^	%	n^P^
*Interventions legitimized by the Finnish Mental Health Act*			
Seclusion	63	15.8	29
Intramuscular medication administered without consent	15	3.8	11
Limitation of the freedom of movement	13	3.3	12
Mechanical restraint	9	2.3	5
Physical restraint	3	0.8	3
Limitation of contacts	3	0.8	3
*PRN-medication per os*	134	33.5	49
*Focused discussion performed by a nurse*	43	10.8	32
*Other interventions*			
Verbal enforcing of boundaries	25	6.3	17
Change of medication	23	5.8	20
Discharge or transfer to another ward	14	3.5	14
A visit by a relative	11	2.8	10
Daily activities/sports/a walk outdoors	11	2.8	11
Leading the patient to her/his own room	10	2.5	6
The patient is held by the arm and purposefully guided away from one location to another	5	1.3	5
The patient stays voluntarily in the seclusion room with the door unlocked	4	1.0	4
Discussion with a doctor	3	0.8	3
Changing the patient room	3	0.8	3
Intramuscular medication with consent	2	0.5	1
A visit to home	2	0.5	2
Giving a structural self-assessment instrument (e.g., the Beck Depression Inventory) to be filled in by the patient	2	0.5	2
Medication given earlier than prescribed	1	0.3	2
Discussion with a social worker	1	0.3	1

*PRN-medication* pro re nata, medication given as needed, *N* ^*I*^ number of interventions, *n* ^*p*^ the number of individuals, who were targeted with this intervention

### Impact of interventions

Multilevel linear regression modelling showed that only the category of “other interventions” (see Table 2) was significantly associated with a lower DASA total score on the following day, and this was observed when examined separately (B = −0.70; CI: −1.24, −0.16; *p* = 0.01) or when adjusted for the use of other concurrent interventions (B = −1.07; CI: −1.17, −0.05; *p* = 0.03). Interventions regulated by the Finnish Mental Health Act, PRN-medication, and focused discussion with nurse were not significantly associated with a lower DASA total score on the following day (Table 3).

**Table 3:** Intervention’s effect on the following day’s Dynamic Appraisal of Situational Aggression (DASA) total score

	Separate associations^a^			Mutually adjusted associations^b^		
Intervention group	B	CI (95%)	*p*	B	CI (95%)	*p*
Interventions regulated by the Finnish Mental Health Act	0.25	−0.35; 0.86	0.41	0.27	−0.34; 0.87	0.38
PRN-medication	−0.44	−1.00; 0.11	0.12	−0.34	−0.91; 0.23	0.24
Discussion with a nurse	−0.32	−0.98; 0.34	0.34	−0.08	−0.75; 0.60	0.82
Other interventions	−0.70	−1.24; −0.16	**0.01**	−1.07	−1.17; −0.05	**0.03**

*PRN-medication* pro re nata, medication given as needed, *Cl* confidence interval. Multilevel linear regression modelling was used for analysis. ^a^The model was adjusted for the baseline DASA score on the intervention day and each intervention was assessed in separate regression models. ^b^The model was adjusted for all interventions and the baseline DASA score on the intervention day. The statistically significant differences are presented in bold

## Discussion

As far as the authors are aware, this is the first study to investigate the use of interventions following a high score in DASA. Approximately 20% of the inpatients on the admission ward scored high on violence risk assessment at least once during their treatment periods. In most cases this led to intervention/s aiming to reduce perceived violence risk. The most frequently used intervention was PRN-medication (33.5%). It was also used alone without any other intervention method in 15.7% of the cases. Previous studies have shown that PRN-medication is frequently used in psychiatric care for various reasons, mainly to decrease patient agitation, although there seem to be only limited evidence for its effectiveness [26, 27]. In the present study, PRN- medication was not significantly associated with on the following day’s DASA total score, implying that it did not decrease the perceived risk of violence. It has been stated that the current use of PRN- medication is based on clinical experience and habit rather than high quality evidence of real pharmacological effect [28]. The use of PRN-medication often lacks a clear chain of accountability and nurses are reluctant to increase their responsibilities for assessing the need for extra medication [29]. There is, however, evidence that consumption of PRN-medications can be reduced with slight changes in ward policies without disadvantages [30].

Another commonly used intervention was seclusion. The use of restrictive methods has been widely debated [4] and various attempts have been made to reduce the use of seclusion and restraint in psychiatric nursing [31–33]. The use of coercive methods has been reported to impair the therapeutic alliance [4, 34], as patients often consider the use of coercive methods as a form of punishment [35, 36] and see them as negative interventions [34, 37]. The high usage rates of restrictive methods reflect the culture of Finnish psychiatric care, and during recent years, the national policy has been to reduce the use of coercion in psychiatric care [38]. Interestingly, nurses have also reported to often feel relieved after the seclusion is over and concerned whether they did the right thing or failed to find alternative methods [33]. Researchers still disagree whether decreasing the use of seclusion results in increasing of violent incidents [39] or whether it has no effect [40] or a decreasing effect [41].

The third most often used intervention was focused discussion with a nurse, which is the nursing intervention used in everyday clinical practice as a first step alternative to seclusion and restraint [31]. Patients, nurses and physicians have all stressed the importance of regular discussions with the patients, especially those between the patient and her/his primary nurse [33, 36, 42]. Empathetic listening, attention and understanding, active communication, a mindful presence here and now, tactfulness, and humane reflection on the patient’s psychopathology are essential elements of focused discussions [33, 42]. A nurse being present and discussing with her/his patient creates an atmosphere of safety, comfort and understanding, but, in the same time, gives the nurse a better understanding of the current mental state of the patient [33, 42]. Focusing on reasons for aggressive behavior can facilitate better ways of dealing these problems on the ward [31]. It is worth noting that nurses have described difficulties in discussing about violence and a threat of it with their patients [20], so active training and support is needed for this highly sensitive topic.

Other interventions such as daily activities were not as frequently used as the above-mentioned ones. Nevertheless, they turned out to be the intervention type significantly associated with the decrease of the perceived risk of violence, and the use of purposeful activities have been reported to reduce the use of both seclusion, restraint and PRN-medication [43, 44]. It is worth noting that discussion with a psychiatrist was mentioned as an intervention only three times during the whole 6-month study period, although there were two psychiatrists working on the ward. It appears that psychiatrists are so overladen by other consecutive duties that no time remains to participate in the preventive actions on the ward. Not once was an appointment with a psychologist or occupational therapist mentioned as an intervention, and discussion with a social worker was mentioned only once. The results imply that nurses can consult supplemental staff if needed, but the special workers mainly remain to be outsourced from situations where there are threats of violence. There is a constitutional need to reconsider the policy of special workers’ abilities to participate in the ward’s everyday routines. Attendance with patients should be increased to identify the potentially aggressive incidents and to integrate all parts of the multi-professional team as part of the ward environment.

### Strengths and limitations

The data were collected from a regular Finnish admission ward and comprised a consecutive sample of inpatients. Nurses on duty had performed the DASA assessments as a team, thus they represented consensus statements rather than the opinions of individual staff members. Comprehensive coaching and a pilot phase had preceded the data collection. None of the researchers worked on the ward, therefore the study represents an objective evaluation. Some study limitations need, however, to be considered when interpreting the current findings. First, the interventions were collected from patients’ medical files. The quality of the medical files varied, and it is possible that occasionally an intervention had occurred even it was not recorded. In Finland, use of coercive methods based on the Finnish Medical Act and medication must be marked down on patients’ medical files by the law in order to be further reported to the local Regional State Administrative Agency, which supervises the proceedings of all Finnish psychiatric hospitals. This does not concern non-coercive methods, which may distort the real picture of the interventions used. Second, only one ward from one hospital was included in the study, and therefore the generalizability of the results may be questioned. Third, the psychiatric diagnoses were not based on structured interviews, but were taken from patients’ medical files. In this regard, in Finland, the basic diagnostic procedures have been proven reliable [45, 46]. Also, the clinical diagnoses were the primary ones, meaning that the possible comorbidity was not taken into account. The length of hospitalization on the Finnish psychiatric admission wards is typically so short that no comorbid diagnoses are assessed, which was the case also at the present ward. However, in this study, the diagnoses were used only as background variables. Further, the study was an observational one in natural setting without any randomization. Therefore, the observed changes in DASA total scores associated with different interventions may have been confounded by unmeasured variables and by indication bias. Indication bias may have arisen if less severe violent incidents were treated with less intrusive interventions and more severe incidents with more coercive methods, and if the less severe incidents represented more temporal and short-term aggressive behavior than the severe incidents. The analysis was adjusted for the baseline DASA total score, which removes some of the confounding, resulted by multiple DASA scores from the same patient (median 3), but cannot exclude it completely. In future, cluster randomized trials would be needed to reliably determine the effectiveness of different intervention types. Overall, criticism has been directed towards the use of actuarial methods. It has been suggested that clinicians should rather use the mean and peak values of the previous week’s DASA scores than the latest total score while predicting inpatient aggression [47]. In the present study, we used the latest total DASA scores, as most of the patients were on ward only some days (median 4). Unfortunately, no internationally acknowledged structural instrument for monitoring aggressive incidents (like the Staff Observation Aggression Scale [48]) was used on the ward. Thus, we were not able to study the impact of interventions on the violent events. Data from the Hospital District’s Risk Report System from the study period served only as a background variable, reflecting the nature of the study ward, as the data in the risk report system is anonymous and cannot be combined with the data gathered from patient records. Further studies focusing on associations between risk-reducing interventions and violent events are obviously needed to enlighten the causality between interventions and violence reduction.

## Conclusion

In most cases, identification of a high risk patient led to an intervention. Unfortunately, the nature of the most frequently used methods was psychopharmacological or coercive. Non-coercive and non-pharmacological interventions like daily activities associated significantly with the decrease of perceived risk of violence, encouraging psychiatric staff to use their imagination when choosing intervention techniques.
